# Supplementary material for: Identification of Freezing-Responsive microRNAs and Their Targets in Chinese Jujube by Small RNA and Degradome Sequencing
Source: Int J Mol Sci. 2026 Sep 4;27(17):7912. doi: 10.3390/ijms27177912 (PMC13566890; doi:10.3390/ijms27177912)
Supplement: Supplementary file 1 [file ijms-27-07912-s001.zip › Supplementary Material S2/novel_miR_211.pdf]

Provisional ID : novel_miR_211
Score total : 900.9
Score for star read(s) : 3.9
Score for read counts : 894.4
Score for mfe : 1
Score for randfold : 1.6
Score for cons. seed :
Total read count : 1766
Mature read count : 1748
Loop read count : 0
Star read count : 18

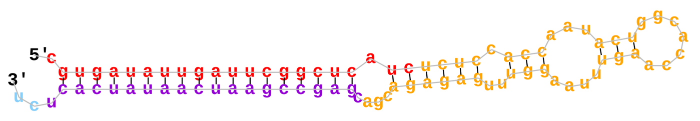

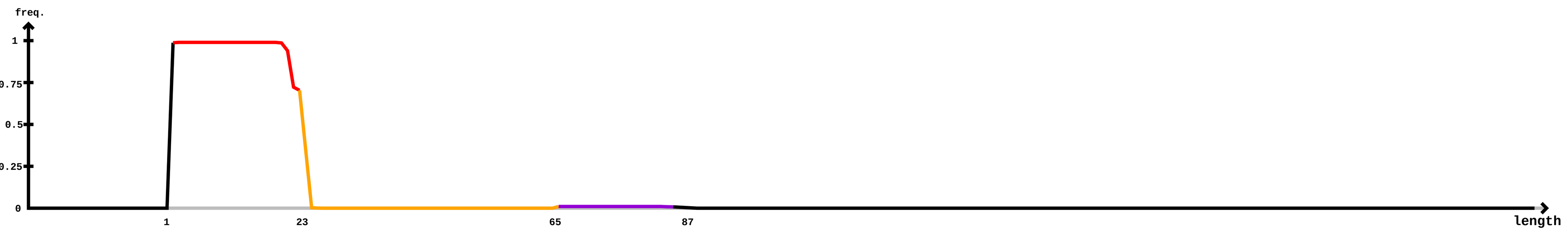

Mature Star

```
5'- cauucugcagaaguagacauggcgugauauugauucggcucaucucuccaccaauacuggcaccaaguuuaagguuugagagacgacgagccgaaucaauaucacucuuguaugcuucuucgcauauuaucaagguuuuuccuguuuuuugaguuccaauauuagccaagaagcaucucugcaggcaacucaugcauuaagcuacauagaagcuuguuuuggcucuccaucccccucuuguaaagucuuc  -3'  obs
    cauucugcagaaguagacauggcgugauauugauucggcucaucucuccaccaauacuggcaccaaguuuaagguuugagagacgacgagccgaaucaauaucacucuuguaugcuucuucgcauauuaucaagguuuuuccuguuuuuugaguuccaauauuagccaagaagcaucucugcaggcaacucaugcauuaagcuacauagaagcuuguuuuggcucuccaucccccucuuguaaagucuuc       exp
    ....((((((.........((.(((((((((((((((((.((((((.(((...(((.......)))....)))..))))))....)))))))))))))))))).)).((((((......))))))......((.....))(((((((((.(((........))))))))))))..)))))))(((.....)))..((((((.......))))))....((((...((.........))...))))...  reads  mm  sample
    ......................cgugauauugauucggcu..........................................................................................................................................................................................................  1    0   LT-1
    ......................cgugauauugauucggcu..........................................................................................................................................................................................................  5    0   LT-3
    ......................cgugauauugauucggcuc.........................................................................................................................................................................................................  13   0   LT-3
    ......................cgugauauugauucggcuc.........................................................................................................................................................................................................  21   0   LT-2
    ......................cgugauauugauucggcuU.........................................................................................................................................................................................................  1    1   LT-3
    ......................cgugauauugauucggcuA.........................................................................................................................................................................................................  6    1   LCK-3
    ......................cgugauauugauucggcuc.........................................................................................................................................................................................................  7    0   LT-1
    ......................cgugauauugauucggcuc.........................................................................................................................................................................................................  33   0   LCK-3
    ......................cgugCuauugauucggcuc.........................................................................................................................................................................................................  1    1   LCK-3
    ......................cgugauauugauucggcuca........................................................................................................................................................................................................  60   0   LCK-2
    ......................cAugauauugauucggcuca........................................................................................................................................................................................................  1    1   LT-2
    ......................cgugauCuugauucggcuca........................................................................................................................................................................................................  1    1   LT-3
    ......................cgugauauugauucggcuca........................................................................................................................................................................................................  3    0   JT-3
    ......................cgugauauugauucggcuca........................................................................................................................................................................................................  103  0   LCK-3
    ......................cgugauauugauucggcuca........................................................................................................................................................................................................  1    0   JT-2
    ......................cgugauCuugauucggcuca........................................................................................................................................................................................................  1    1   LCK-2
    ......................cgugauCuugauucggcuca........................................................................................................................................................................................................  1    1   LCK-1
    ......................cgugauauugauucggcuca........................................................................................................................................................................................................  9    0   JCK-2
    ......................cgugauauugauucggcuca........................................................................................................................................................................................................  3    0   JCK-3
    ......................cgugauauugauucggcucU........................................................................................................................................................................................................  3    1   LT-2
    ......................cgugauauugauucggcuca........................................................................................................................................................................................................  23   0   LT-1
    ......................cgugauauugauucggcuca........................................................................................................................................................................................................  48   0   LCK-1
    ......................cgugauauugauucggcuca........................................................................................................................................................................................................  2    0   JCK-1
    ......................cgugauauugauucgAcuca........................................................................................................................................................................................................  1    1   LCK-3
    ......................cgugauauugauucggcuca........................................................................................................................................................................................................  52   0   LT-3
    ......................cgugauauugauucggcuca........................................................................................................................................................................................................  71   0   LT-2
    ......................cgugauauugauucggcucau.......................................................................................................................................................................................................  14   0   LCK-3
    ......................cgugauauugauucggcucau.......................................................................................................................................................................................................  1    0   LT-3
    ......................cgugauauugauucggcucau.......................................................................................................................................................................................................  8    0   LT-1
    ......................cgugauCuugauucggcucau.......................................................................................................................................................................................................  1    1   LT-3
    ......................cgugauauugauucggcucau.......................................................................................................................................................................................................  9    0   LT-2
    ......................cgugauauugauucggcucaA.......................................................................................................................................................................................................  1    1   LT-3
    ......................cgugauauugauucggcucauc......................................................................................................................................................................................................  51   0   JCK-1
```

Mature **Star**

cauucugcagaaguagacauggcgugauauugauucggcucaucucuccaccaauacuggcaccaaguuuaagguuugagagacgacgagccgaaucaauaucacucuuguaugcuucuucgcauauuaucaagguuuuuccuguuuuuugaguuccaauauuagccaagaagcaucucugcaggcaacucaugcauuaagcuacauagaagcuuguuuuggcucuccaucccccucuuguaaagucuuc			
......................Agugauauugauucggcucauc..	1	1	LT-1
......................cgugauauugauucggcucauc..	137	0	LCK-3
......................cgugauauugauucggcucauU..	1	1	JCK-1
......................cgugauauugauucggcucauU..	5	1	LT-3
......................cgugauauugauucggcucauc..	122	0	LT-2
......................cgugauauugauucggcucauU..	13	1	LT-2
......................cgugauauugCuucggcucauc..	1	1	LT-1
......................cgugauauugauucggcucauU..	5	1	JCK-3
......................cgugauauugauucggcucauc..	99	0	JCK-2
......................cgugCuauugauucggcucauc..	2	1	JCK-2
......................cgugauauugauucggcucauc..	31	0	LCK-2
......................cgugCuauugauucggcucauc..	1	1	LCK-3
......................cguUauauugauucggcucauc..	1	1	LT-2
......................cgugauauugauucggcucauc..	31	0	LCK-1
......................cgugauauugauucggcucauc..	93	0	JCK-3
......................cgugauauugauucggcucauU..	1	1	JCK-2
......................cgugauauugauucggcCcauc..	1	1	LT-1
......................cgugauauugCuucggcucauc..	1	1	JCK-2
......................cNugauauugauucggcucauc..	1	1	JCK-1
......................cgugauauugauucggcucauU..	3	1	LT-1
......................cguAauauugauucggcucauc..	1	1	JCK-3
......................cgugauauugauucggcucauc..	248	0	LT-3
......................cgugauauugauucggcucauU..	9	1	LCK-3
......................cgugauauugauucggcucCuc..	1	1	LT-2
......................cgGgauauugauucggcucauc..	1	1	LT-2
......................cgugauauugCuucggcucauc..	1	1	JCK-1
......................cgugauauugauucggcucauc..	237	0	LT-1
......................cgugCuauugauucggcucauc..	1	1	LT-3
......................cgugauauugauucggcucaucA...	1	1	LCK-3
......................cgugauauugauucggcucaucu...	3	0	JCK-2
......................cgugauauugauucggcucauAu...	1	1	LT-2
......................cgugauauugauucggcucauUu...	9	1	LCK-3
......................cgugauauugauucggcucauUu...	6	1	LT-2
......................cgugauauugauucggcucaucA...	2	1	LT-1
......................cgugauauugauucggcucaucu...	17	0	LT-2
......................cgugauauugauucggcucauUu...	1	1	LT-3
......................cgugauauugauucggcucaucu...	4	0	JCK-3
......................cgugauauugauucggcucaucu...	30	0	LT-1
......................cAugauauugauucggcucaucu...	1	1	LT-2
......................cgugauauugauucggcucaucu...	15	0	LCK-3
......................cgugauauugauucggcucauUu...	2	1	LT-1
......................cgugauauugCuucggcucaucu...	1	1	LT-1
......................cgugauauugauucggcucaucu...	1	0	JCK-1
......................cgugauauugauucggcucaucu...	34	0	LT-3
......................cgugauauugauucggcucaucA...	3	1	LT-2
......................cgugauauugauucggcucaucA...	3	1	LT-3
......................cgugauauugauucggcucaucuU..	2	1	LT-3
......................cgugauauugauucggcucaucuU..	1	1	JCK-2
......................cgugauauugauucggcucaucuU..	1	1	LT-2
......................cgugauauugauucggcucaucuUu...	1	1	LCK-3
.......................gugauauugauucggcuca..	1	0	LT-2
.......................gugauauugauucggcucauc..	1	0	LT-1
.......................gugauauugauucggcucauc..	2	0	LT-3
....cgagccgaaucaauaucU..	2	1	LCK-3
....cgagccgaaucaauauca..	1	0	LT-2
....cgagccgaaucaauaucac...	1	0	LT-1
....cgagccgaaucaauaucacu..	6	0	LT-1
....cgagccgaaucaauaucacu..	2	0	LT-3
....cgagccgaaucaauaucacu..	5	0	LT-2
....cgagccgaaucaauaucacuU...	1	1	LT-1
